# Facile fabrication of complex networks of memristive devices

**DOI:** 10.1038/s41598-017-08244-y

**Published:** 2017-08-11

**Authors:** Chloé Minnai, Andrea Bellacicca, Simon A. Brown, Paolo Milani

**Affiliations:** 1 CIMAINA and Dipartimento di Fisica, Università degli Studi di Milano, via Celoria 16, 20133 Milano, Italy; 2 The MacDiarmid Institute for Advanced Materials and Nanotechnology, Department of Physics and Astronomy, University of Canterbury, Private Bag 4800, Christchurch, 8140 New Zealand

## Abstract

We describe the memristive properties of cluster-assembled gold films. We show that resistive switching is observed in pure metallic nanostructured films at room temperature and atmospheric pressure, in response to applied voltage inputs. In particular, we observe resistance changes up to 400% and archetypal switching events that have remarkable symmetry with the applied voltage. We associated this symmetry with ‘potentiation’ and ‘anti-potentiation’ processes involving the activation of synapses and of pathways comprising multiple synapses. The stability and reproducibility of the resistance switching, which lasted over many hours, make these devices ideal test-beds for exploration of the basic mechanisms of the switching processes, and allow convenient fabrication of devices that may have neuromorphic properties.

## Introduction

The approaching end of Moore’s law as the compass for the technology roadmap of the semiconductor industry^1^ has prompted a change of strategy towards the integration of different capabilities such as sensing, actuation, power management, data storage, and remote connectivity on the same device^2^. This requires the fabrication of platforms where the integration of different materials and functionalities is the enabling factor, and miniaturization is not the major issue. Among different strategies for the fabrication of this novel class of hybrid devices, the combination of top-down fabrication with bottom-up synthetic methods appears very promising^3^. In particular, devices obtained by the bottom-up assembly of atomic clusters on microfabricated platforms leads to functional properties that are promising for a wide spectrum of applications^4^.

The same considerations are also affecting the way in which computing architectures and data storage systems are integrated on complex platforms. A new approach for computer architectures aims to achieve low-power consumption by using brain-like neuromorphic systems that are characterized by high parallelism and network structures that are able to process information very efficiently^5–12^. One of the most promising architectures^5, 11^ is based on an assembly of interconnected nanoscale switching elements that exhibit synapse-like behaviour^11, 13, 14^. The elemental building blocks of these kinds of networks are memristors^14–16^ which are nonlinear circuit elements that change their resistance depending on the history of bias applied to them^17^. Memristive behaviour is associated with electrical and structural changes caused by the displacement and rearrangement of mobile ions or oxygen vacancies, the formation and rupture of conductive paths or phase transitions^16, 18–20^.

Fabrication using standard lithographic techniques such as those used for CMOS technology or for two-terminal planar memristors^8, 21, 22^ is costly and complicated due to the need to deterministically create robust intra- and inter-device connections. There has been significant progress in the emulation of neurons and synapses using CMOS circuitry^8, 9^ but the prospect that the same functionality could be achieved from networks of neuron-like and synapse-like elements that are randomly assembled from nanoscale components is very appealing since such biomimetic architectures could potentially by-pass fundamental bottlenecks and cost constraints^9, 11^. Bottom-up random assembly of nanoscale building blocks has been proposed as a cheaper and simpler alternative route to fabrication of neuromorphic networks of memristive devices^11^. To create operable networks two basic issues must be addressed: which materials to use and how to pattern them into complex configurations without negatively affecting their functional characteristics.

Metal nanowires and nanoparticles are considered very interesting candidates to solve these issues - for example networks of sulphidised silver nanowires can be used to achieve simple pattern recognition^5, 11^. In a similar alternative approach cluster-assembled percolating networks exhibit interesting switching behaviour^23^ and numerical simulations^24^ show that they may exhibit potentiation in response to applied voltage inputs. Potentiation is a characteristic of biological systems like the brain that comprise neurons and synapses, and results from activation of a number of synapses to produce a connected pathway across the system^25^. In a simplified form such a connection represents learning by the system from its inputs (a memory). These ideas can be seen as aligning with a wider interest in neuromorphic behaviour^15, 26, 27^.

The assembling of atomic clusters produced in the gas phase has reached a high degree of maturity and it can be considered an enabling technology for the large-scale fabrication of devices for sensor and biomedical applications^4, 28, 29^. Cluster-assembled materials possess a nanostructure that derives from the individual clusters and which follow universal scaling laws^30, 31^. The electrical properties at the percolation threshold of assemblies of metallic nanoparticles and nanoislands have been subjects of intense investigation as a particular class of inhomogeneous conductors^23, 32, 33^. Percolation phenomena play a major role in the metal-insulator transitions observed in many such disordered and composite systems^34, 35^, and in the anomalous conductivities of ultrathin metallic films^36–38^.

Here we present the facile fabrication of cluster-assembled gold nanostructured films that exhibit memristive switching properties. The networks are prepared by supersonic cluster beam deposition (SCBD)^39^ on standard glass substrates at room temperature of nanoparticles with a density close to the electrical percolation threshold. Detailed electrical characterisation reveals complex sequences of switching events and we show that the individual switching events can be characterised according to 3 main archetypes. The films exhibit remarkably reproducible switching behaviour and we show that switching mechanism is based on the formation and destruction of atomic-scale conductive paths.

## Results

During the cluster deposition the device resistance is observed to drop approximately exponentially, consistent with a reduction in the size of tunnelling gaps between the particles as the film coverage increases^24, 40^. By monitoring the resistance it is possible to terminate the deposition when the cluster density is close to the percolation threshold and the resistance is in the range of interest (1 kΩ to 1 MΩ).

**Figure 1:**
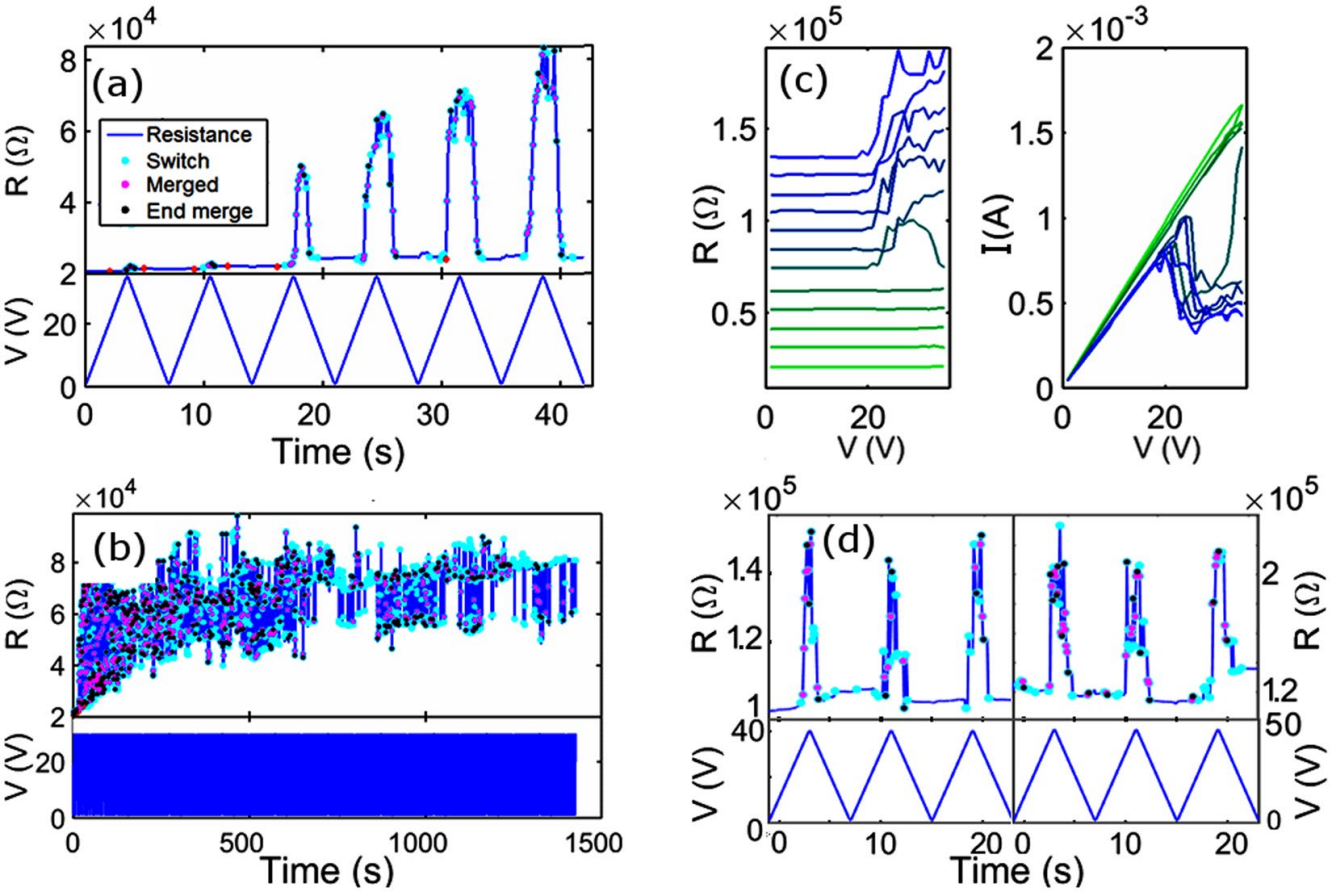
Switching events in SCBD Au-glass thin film. **(a)** Detailed view of initial switching events. Each event is labelled with a coloured symbol. Top: the resistance changes as a function of time (R(t)). Bottom: the voltage ramp (V(t)), in this case between 0 and V_max_ = 40 V. **(b)** Much longer switching sequence; the first 40 s corresponds to the data in (**a**). **(c)** R(V) and I(V) curves corresponding to switching events reported in (**a**). R(V) curves are offset for clarity. The first voltage ramps are shown in bright green and the last in deep blue. Alternate curves correspond to increases and decreases in voltage. **(d)** Comparison between the switching behaviour of the devices near the end of the first (left panel) and second (right panel) days of measurements.

By applying voltage ramps (to a maximum voltage, V_max_) to the cluster-assembled films we observe the onset of complex switching behaviour, with individual switching events resulting in discrete changes in the measured resistance (see Fig. 1 and the Supplementary Information). Initially, at low V_max_ ~ 10 V, no switching is observed for any of the films. As V_max_ is increased beyond 10 V, we observed two different types of behaviour. If the device’s initial resistance is lower than about 10 kΩ, the voltage ramping causes a continuous gradual increase in the resistance. No switching events occur even for V ~60 V. On the other hand, samples with a resistance higher than 10 kΩ exhibit a well-defined voltage threshold beyond which a complex cascade of switching events occurs. V_threshold_ increases with the initial resistance of the sample: 30–40 V is enough to activate samples with an initial resistance in the range 10–20 kΩ, whilst 90–100 V is necessary to activate samples with a resistance in the range 60–70 kΩ. We focus here on the most common, archetypal switching events that were recorded thousands of times in all devices fabricated with a resistance higher than 10 kΩ. Samples with different densities of nanoparticles and hence with lower and higher resistances have been also produced and characterized as reported in the Supplementay Informations (Fig. S1).

Figure 1 shows a typical switching history of a cluster-assembled film with an initial resistance of ~20 kΩ in the first few voltage ramps (Fig. 1(a)) small switching events, hardly visible on this scale, are observed near V_max_, then (3rd voltage cycle) much more dramatic switching is observed. In each cycle a cascade of events is observed during the increase in voltage, as discussed later, this behaviour is similar to the potentiation process discussed in ref. 28. The remarkable feature is that when the voltage decreases there is an almost exact reversal of the sequence: the resistance decreases so that the final resistance at the end of each voltage ramp (when the voltage returns to ~0 V) is very similar to the initial resistance. In the 6th cycle the cascade of switching events results in a change of resistance of ~80 kΩ, an increase of ~400%.

For each device, we continued ramping the voltage to the same V_max_ until it was established that the switching was reproducible over many cycles, or until the number of events diminished. In the latter case, it was almost always found that the switching was reactivated when V_max_ was increased by a few volts. We observe that right after the activation, and when V_max_ is increased, the switching rate and amplitude increase. Subsequently the sample resistance stabilizes at a slightly higher value, and the switching effect (amplitude and rate) returns to values which are then consistent for days - see Fig. 1(d). This stabilization process is particularly evident right after the activation (see Fig. 1(b)), another example is shown in Fig. S1. This behaviour is typically observed for many hours up to several days. Note that the device resistance remains in a well-defined range over many hours of continuous measurements (Fig. 1(b)). In Fig. 1(d) we show that the devices exhibit a similar switching behaviour over periods of days: typical data towards the end of the first and second days of measurements are shown in the left and right panels respectively.

For the sake of completeness, we report in Fig. 1(c) the I(V) and R(V) curves corresponding to the data shown in Fig. 1(a). Obviously, the increase in resistance in each cycle corresponds to the decrease in current at higher voltages in each I(V) curve. I(V) and R(V) curves are typically used to demonstrate memristive behaviour ^18, 19^ but in the present case we believe R(t) curves are a more effective way of showing the observed switching behaviour, and all the data presented below is shown in this form.

**Figure 2:**
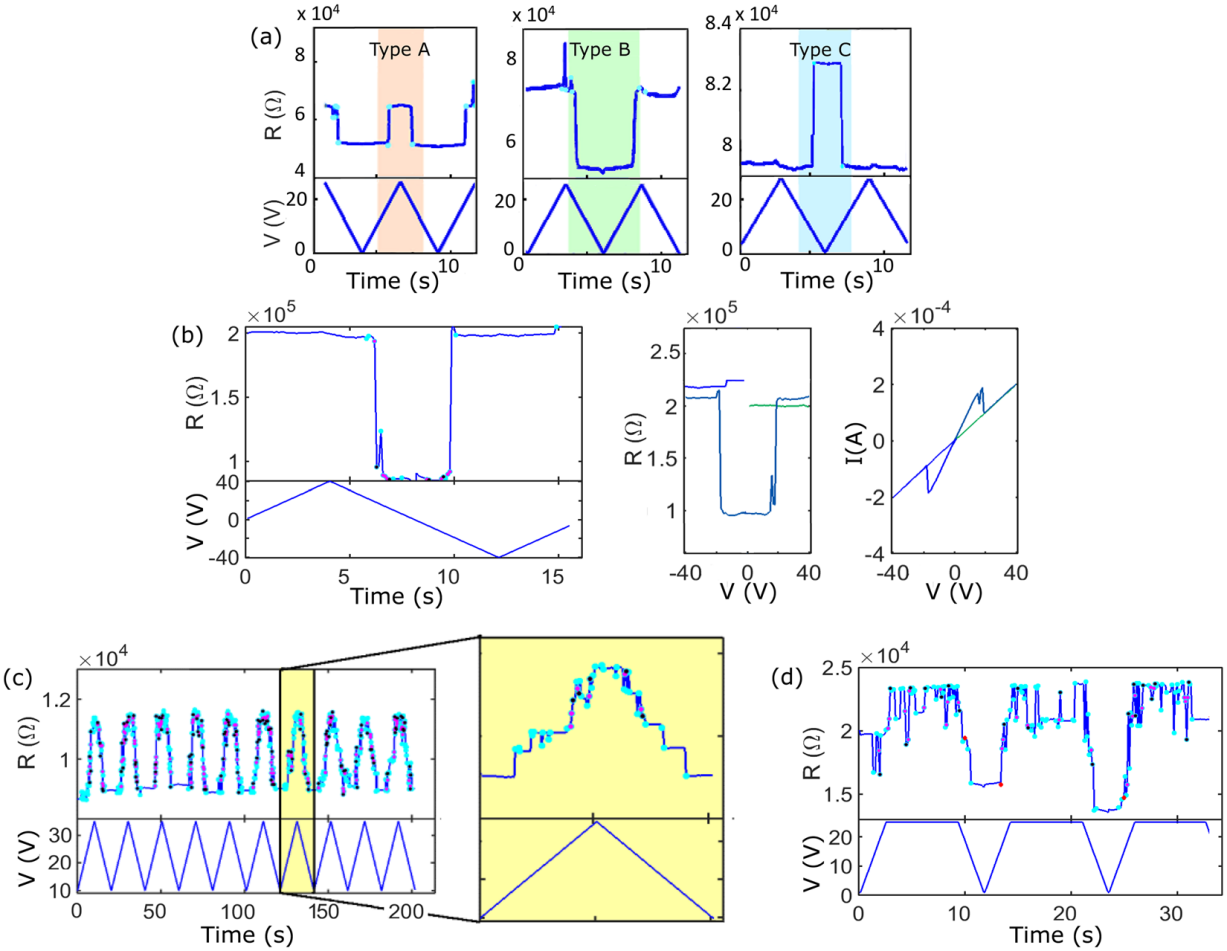
Examples of characteristic switching events. **(a)** The three simplest types of events are reported: resistance increase at high voltage (pink region); resistance decrease at low voltages (green region); and resistance increase at low voltage (blue region). These archetypes are referred to as Type A, B and C respectively. **(b)** Example of bipolar switching occurring while the voltage is ramped between positive and negative V_max_ values. The R(t), R(V) and I(V) curves are shown. **(c)** Examples of highly reproducible sequences of multiple overlapping switching events on consecutive cycles resulting in ‘anti-potentiation’; in the inset: multiple well-resolved events resulting in a ‘Mayan pyramid’ R(t) profile. **(d)** Switching events recorded at high voltage.

Each device exhibits a complex sequence of switching events (as in Fig. 1), but when V_max_ ~ V_threshold_ the switching is less complex and archetypal events can be identified, as shown in Fig. 2.

In Fig. 2(a), the pink region highlights a typical event in which a stepwise increase in resistance is observed while increasing the voltage (Type A event), and the green region highlights a stepwise decrease in resistance that occurs while decreasing the voltage (Type B event). In both cases, the resistance change is reversed by a single stepwise jump at a comparable voltage (typically ~ 10 V) on the subsequent voltage ramp. In both cases the device changes state from a low voltage, low resistance ‘ON’ state to a high voltage, high resistance ‘OFF’ state.

One of the remarkable features of these transitions is their symmetry i.e. the transitions occur at almost exactly the same voltage during the increasing and decreasing voltage ramps and the measured resistance returns to its original value. This means that a connection breaks when the voltage is at its high level and *the same* connection is restored when the voltage is at a low level. Spikes in resistance near V_max_ are also commonly observed (e.g. at the left edge of the green region in Fig. 2(a)); we believe that these are examples of Type A events that last for a shorter time.

A further type of switching event is shown in the blue panel of Fig. 2(a). In these Type C events there is a symmetrical increase in resistance at *low* voltage. Note that these events are different from those of the Type B where the increase in R occurs at high voltages. Type C events clearly have a different physical origin to Type A and B events and are due to destruction of connections as the voltage is reduced, or, completely equivalently, the formation of connections as the voltage is increased^23^.

The data presented so far were obtained with unipolar voltage ramps. Further information is obtained from *bipolar* voltage ramps i.e. voltage ramps in which consecutive cycles have opposite polarities i.e. + V_max_ and –V_max_. Figure 2(b) shows R(t), R(V) and I(V) curves for bipolar switching to a low voltage, low resistance state similar to the Type B events. Note however that in these cases the symmetric switching events happen at the same voltage (i.e. |V|) but with *opposite* sign. Hence the switching mechanism is polarity *independent*.

Figure 2(c) shows a typical dataset in which multiple switching events are observed on each voltage cycle. Events are observed only above a threshold voltage and the device resistance returns to a value close to the original one when the voltage drops below the threshold. In each cycle there is a cascade of switching events which generally increase the resistance, although the resistance commonly switches back and forth a few times between the new level and the previous level. When the voltage is decreased the pattern of switching is reversed until, below the threshold, the resistance returns to a value close to its original value.

The inset of Fig. 2(c) (shaded yellow) shows that the resistance values measured while increasing the voltage are similar to those measured during the decreasing voltage. This “Mayan pyramid” profile results from a series of consecutive Type A events and suggests strongly that the same states of the switches are accessed on both increasing and decreasing voltage ramps. Similar cascades are observed on many consecutive voltage ramps with the resistance always returning to a value close to its original value.

It can also be noticed in Figs 1(a) and 2(c) (inset), that more events occur when the voltage is close to V_max_. This is even more evident in Fig. 2(d) where during each ramp, the voltage is kept constant for some time at the V_max_ value. A cascade of events is observed when the voltage is maximum, suggesting that the connections are continuously broken and re-formed.

## Discussion

**Figure 3:**
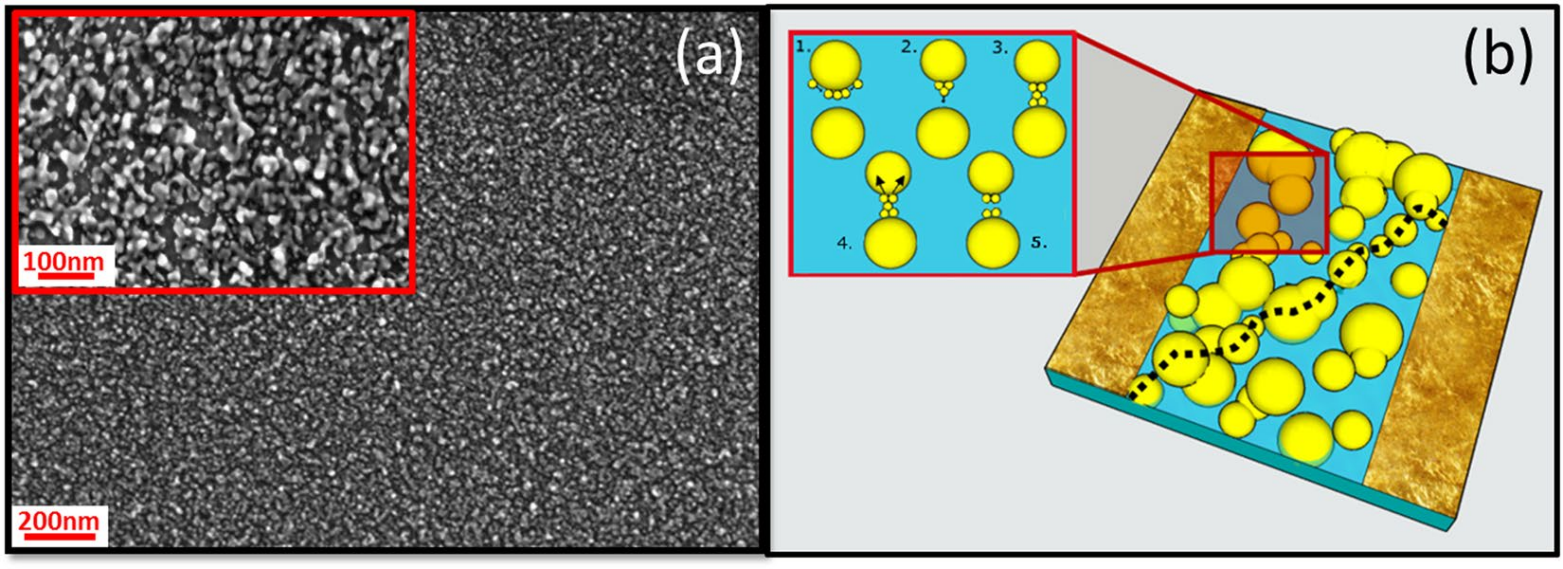
SCBD Au-Glass thin film. **(a)** Au clusters deposited on glass substrate with SCBD acquired with a Field Emission Scanning Electron Microscopy (FEG-SEM). **(b)** Schematic illustration of a percolating-tunneling system. A tunneling path is marked with a dashed line. In the inset the main phenomena which can lead to the formation of an atomic scale wire in a tunnel gap are depicted: 1. EFISD or van der Waals forces; 2. EFIE process; 3. Connected atomic wire. 4. Electromigration affecting the atomic wire: 5. Breaking of the atomic wire due to electromigration.

SEM images of the nanostructured films (Fig. 3(a)) clearly show a complex structure resulting from the random assembling of clusters to form a non-continuous film: both connected particles and tunnel gaps can be distinctly observed, as shown schematically in Fig. 3(b) where, for sake of simplicity, complex aggregated structures have been replaced with large spherical particles. We believe that the mechanism underlying the observed complex switching phenomena can be related to the formation, growth and breaking of junctions between the particles forming the films. A similar model was used to explain the formation of atomic scale wires in percolating films of Sn nanoparticles^23^.

In the inset of Fig. 3(b) a sequence of cartoons shows the mechanism leading to atomic wire formation (inset, sequence 1–3) and destruction (inset, sequence 3–5). The mechanism of formation or growth of connections between adjacent grains may be similar to that which is responsible, in the macroscopic case, for the Branly effect^34, 41, 42^. There, the effect is usually explained by diffusion of atoms at an already existing junction or gap and by Joule heating, but we believe that Joule heating alone cannot be responsible for switching since in our experience it causes local destruction of the films that cannot be reversed.

We believe that the strong electric field within the tunnel gaps in the film leads to Electric Field Induced Surface Diffusion (EFISD) or Electrical Field Evaporation (EFIE) (inset, 2) and that attractive van der Waals forces (which are independent of applied electric field) can also drive formation of atomic scale connections. In the case of non-connected grains, as the gap reduces, the electric field become stronger, causing the formation of an atomic scale wire (inset, 3). EFISD, EFIE and van der Waals forces can all create new pathways between the particles. On the other hand, the high electrical currents within the reorganized junctions and the newly formed connections, cause electromigration^22^ (inset, 4) and, as a consequence, the breakdown and the disconnection of the path. We believe these processes are at the origin of the switching events reported so far. In particular, for both Type A and B events, the application of a voltage tends to increase the resistance of the network by breaking pre-existing connections between particles in the film via electromigration (inset, sequence 3–5).

Physically, Type A and Type B events have the same origin: a high resistance at high voltage is due to broken connections and at low resistance at low voltage is due to the formation of connections in the film. However, events that result in a high resistance at high voltage (Type A events) are caused when an established connection is broken by the high current that is flowing, resulting in a higher resistance. In Type B events the decrease in resistance during the decreasing voltage ramp is due to formation of a new atomic scale wire, which is broken at a comparable voltage on the next increasing voltage ramp.

The subsequent decreases in resistance occur due to reconnection across those gaps, which are most likely due to van der Waals forces^23, 43^: the wire appears to be formed at voltages below some threshold (typically 10–30 V) because the current that is flowing is no longer sufficient to break the wire. At higher voltages, the wire cannot reform because electromigration instantly breaks it. This argument is also supported by the data shown in Fig. 2(d). Here, when the voltage is kept constant at the maximum value of 25 V, the connections are continuously being broken (causing increases in resistance) and then re-forming (causing the resistance returning to its original value). In this model the state of the network is dynamic, and the observed resistance is determined by the complex interplay of events that break and re-form individual atomic scale wires.

The remarkable symmetry of the Type A and B events indicates that the apparent threshold voltage for switching is the voltage at which the interplay between van der Waals forces and electromigration are equally balanced. Below this voltage van der Waals forces dominate and connections are formed, while at higher voltages electromigration prevails and the connection is broken.

Type C events can be explained with a similar mechanism in which the opposite effects dominate: an increase in voltage causes EFIE or EFISD, forming a connection across the gap between two particles and reducing the device resistance, then, while the voltage is being decreased, the electric current breaks the wire and opens the connection. Hence there is a distinction between electric field driven Type C events and Type A and B events where decreases in resistance are driven by van der Waals forces.

We emphasize again that after a switching event, the resistance returns to approximately its initial value at the end of each voltage cycle. The fact that the same events are repeated on multiple voltage cycles (see for example, Fig. 2(a) and (c)) implies that the *same* connections that are broken while increasing the voltage are re-formed as the voltage is reduced.

The observed features could in principle be explained by other models used to describe similar switching in related systems, but careful examination of the data allows us to eliminate such models. For example, Coulomb blockade (i.e. charging of nanoparticles in the network) can be ruled out by the data from bipolar voltage ramps in Fig. 2(b) since it is demonstrated that the switching behaviour is *insensitive* to the polarity of the applied voltage. In a model of Coulomb blockade, it is inconceivable that charging could consistently occur at a positive voltage and then discharging occurs at an equal and opposite negative voltage. The presence of offset charges in real devices^44, 45^ also means that practically speaking Coulomb charging is an asymmetrical process. A Coulomb charging picture can also be ruled out on more general grounds: refs 44, 46 show that for similar size particles charging effects are expected only at low temperatures and require voltages smaller than ~10 V.

Further alternative switching models that could be considered are those responsible for memristive behaviour in oxide devices, which is typically due to either the motion of defects (typically oxygen vacancies^16, 18^), or to the formation of nanoscale chains of particles due to diffusion of ions in an oxide matrix^21, 47^. Both of these models can be ruled out because in those cases resetting the device state *requires* a change in polarity of the applied voltage (either the oxygen vacancies or the ions must be driven back to their original positions). In contrast, in our devices, the switching behaviour is insensitive to the polarity of the applied voltage. For similar reasons it is possible to rule out electrochemical processes^12^ as being responsible for the switching behaviour since they also require a reversal of polarity to achieve reproducible switching between states.

It is also interesting to notice that the cascade of switching events that lead to resistance *decreases* (e.g. Fig. 2(c)) is very similar to a potentiation process (i.e. the formation of a pathway comprising groups of particles connected synapses) predicted by simulation in refs 23, 28 and the neuromorphic behaviour observed in ref. 5. A remarkable feature of the present data however is that in many cases (for example Fig. 1(a)) the applied voltage first *increases* the resistance of the device. This suggests that each cycle begins with a kind of ‘anti-potentiation’ (breaking of a chain of synaptic connections).

## Conclusions

We have shown that complex networks of memristive switching elements can be fabricated by assembling Au clusters on glass substrates by SCBD. Au clusters are deposited in a ballistic regime producing nanostructured films where the nanoscopic building blocks partially retain their individuality and are connected by grain boundaries and atomic-scale junctions. The observed switching behaviour occurs due to the formation (destruction) of nanoscale connections between particles which occurs in response to high electric fields and van der Waals forces (electromigration).

It is remarkable that this atomic scale switching process yields reproducible switching behaviour over periods of days, even in ambient conditions and using a very simple measurement methodology. These devices therefore provide a convenient test-bed for exploration of the basic mechanism of the switching processes and also the possibility of convenient fabrication of neuromorphic devices^5, 11–13^ comprising large numbers of switching elements.

An important factor that requires further investigation is the role of the network in determining the switching characteristics of the individual elements: both the activity of other switching elements^5^ and the series resistance of the network^22^ can be expected to influence the switching activity. The first steps in evaluating device performance for real-world applications will be to further explore potentiation in these devices as a route towards both processing and storage^24^, and to determine whether these dynamical networks can be used for simple pattern recognition problems^48^.

We remark finally that several architectures for neuromorphic computing have been under development for a number of years, and in some cases (e.g. cross-bar architectures^10, 49^) relatively well-defined specifications have been developed for the required device parameters. A recent review^10^ makes it clear that despite intensive efforts, significant progress is still required for all kinds of neuromorphic devices in order to achieve useful computational performance, and therefore that there is still a need for novel approaches that circumvent the known problems. Target device performance depends significantly on architecture^10^, and is surprisingly varied: for example, high resistances and relatively small percentage changes in resistance are required for computation using some crossbar architectures^49^. Our experiments are consistent with the modelling of percolating networks^28^, in which the individual junctions exhibit high on / off ratios, but our self-assembled devices will need to meet a set of performance criteria whose development will require further detailed modelling and investigations of possible computational algorithms^10, 49^.

## Methods

### Device fabrication

**Figure 4:**
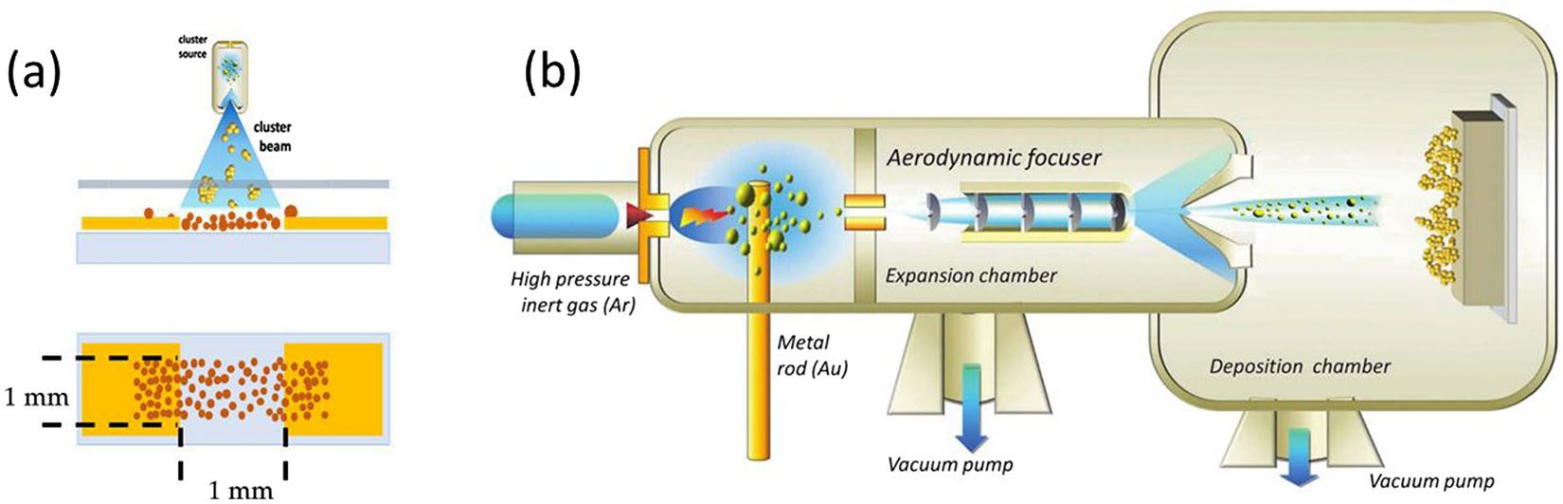
Device fabrication with Supersonic Cluster Beam Deposition. (**a**) Schematic of the cluster-assembled film fabrication. (**b**) Schematic representation of the SCBD apparatus (not to scale).

A schematic representation of the film fabrication steps is reported in Fig. 4(a). Neutral gold clusters are deposited on glass slides in the gap between two gold electrodes previously fabricated by thermal evaporation. The cluster-assembled layer forms a 1 × 10 mm strip partially covering the electrodes. The surface roughness of the glass substrates was measured by AFM to be 1.5 ± 0.3 nm. The gap between the electrodes is 1 mm and the overall device dimension including contact pads is 10 × 10 mm.

Clusters are deposited by means of a SCBD apparatus equipped with a pulsed microplasma cluster source (PMCS)^39^. In Fig. 4(b) a schematic of the apparatus is reported. The PMCS consists of a ceramic body with a cavity in which a gold target is vaporized by a localized electrical discharge supported by a pulsed injection of an inert gas at high pressure. The metal atoms, sputtered from the target, aggregate in the source cavity to form metal clusters; the mixture of clusters and inert gas expands through a nozzle forming a supersonic beam into an expansion chamber kept at a pressure of about 10^−6^ mbar. Electrically neutral nanoparticles exiting the PMCS are aerodynamically accelerated in a highly-collimated beam with divergence lower than 1° and with a kinetic energy of roughly 0.5 eV/atom^29, 39^. The central part of the cluster beam enters, through a skimmer, a second vacuum chamber (deposition chamber) where the beam is intercepted by the substrate. During the cluster deposition, a fixed small voltage (in the range 1–100 mV) is applied to the electrodes in order to monitor *in situ* the evolution of the electrical resistance of the cluster-assembled film.

### Electrical Characterisation

The devices have been electrically characterized under ambient atmosphere by applying voltage ramps and recording the resultant current, hence providing the resistance variation in time. A triangular voltage ramp from 0 V to a maximum voltage, V_max_, is applied, and V_max_ is gradually increased until switching is observed.

## Electronic supplementary material

Supplementary Information

## References

[1] Waldrop M (2016). More than Moore. Nature.

[2] Zhang, G. Q. & Roosmalen A. V. *More than Moore: Creating High Value Micro/Nanoelectronics Systems*. (Springer Science, LLC, 2009).

[3] Curri ML, Comparelli R, Striccoli M, Agostiano A (2010). Emerging methods for fabricating functional structures by patterning and assembling engineered nanocrystals. Phys. Chem. Chem. Phys..

[4] Milani, P. & Bettini, L. G. Nano- and Micromanufacturing with Nanoparticles Produced in the Gas Phase: An Emerging Tool for Functional and Length-Scale Integration, in *Gas-Phase Synthesis of Nanoparticles* (ed. Huttel Y.) (Wiley, 2017).

[5] Avizienis AV (2012). Neuromorphic Atomic Switch Networks. PLoS One.

[6] Yang JJ, Strukov DB, Stewart DR (2013). Memristive devices for computing. Nat. Nanotechnol..

[7] Choi S, Sheridan P, Lu WD (2015). Data Clustering using Memristor Networks. Sci. Rep..

[8] Xia Q (2009). Memristor-CMOS hybrid integrated circuits for reconfigurable logic. Nano Lett..

[9] Ohno, T. in *Micro-and Nanoelectronics: Emerging Device Challenges and Solutions* (ed. Brozek, T.) **35**, 283 (Taylor & Francis Group, 2014).

[10] Burr GW (2017). Neuromorphic computing using non-volatile memory. Adv. Phys. X.

[11] Stieg AZ (2012). Emergent criticality in complex turing B-type atomic switch networks. Adv. Mater..

[12] Ohno T (2011). Sensory and short-term memory formations observed in a Ag2S gap-type atomic switch. Appl. Phys. Lett..

[13] Ohno T (2011). Short-term plasticity and long-term potentiation mimicked in single inorganic synapses. Nat. Mater..

[14] Kim H, Yang C, Chua LO (2012). Memristor Bridge Synapses. in. Proceedings of the IEEE.

[15] Le Doux, J. Synaptic self: How our brains become who we are. (Books, Penguin, 2003).

[16] Prodromakis T, Toumazou C, Chua L (2012). Two centuries of memristors. Nat. Mater..

[17] Chua LO (1971). Memristor—The Missing Circuit Element. IEEE Trans. Circuit Theory.

[18] Strukov DB, Snider GS, Stewart DR, Williams RS (2008). The missing memristor found. Nat. Lett..

[19] Waser R, Aono M (2007). Nanoionics-based resistive switching memories. Nat. Mater..

[20] Gaba S, Cai F, Zhou J, Lu WD (2014). Ultralow Sub-1-nA operating current resistive memory with intrinsic non-linear characteristics. IEEE Electron Device Lett..

[21] Joshua Yang J (2009). The mechanism of electroforming of metal oxide memristive switches. Nanotechnology.

[22] Durkan C, Welland ME (2000). Size effects in the electrical resistivity of polycrystalline nanowires. Phys. Rev. B.

[23] Sattar A, Fostner S, Brown SA (2013). Quantized conductance and switching in percolating nanoparticle films. Phys. Rev. Lett..

[24] Fostner S, Brown R, Carr J, Brown SA (2014). Continuum percolation with tunneling. Phys. Rev. B - Condens. Matter Mater. Phys..

[25] Chialvo DR (2010). Emergent complex neural dynamics. Nat. Phys..

[26] Mead C (1990). Neuromorphic Electronic Systems. Proc. IEEE.

[27] Merolla PA (2014). A million spiking-neuron integrated circuit with a scalable communication network and interface. Science.

[28] Fostner S, Brown SA (2015). Neuromorphic behavior in percolating nanoparticle films. Phys. Rev. E.

[29] Schulte C, Podesta A, Lenardi C, Tedeschi G, Milani P (2017). Quantitative Control of Protein and Cell Interaction with Nanostructured Surfaces by Cluster Assembling. Acc. Chem. Res..

[30] Podestà A (2015). Nanomanufacturing of titania interfaces with controlled structural and functional properties by supersonic cluster beam deposition. J. Appl. Phys..

[31] Dunbar, A. D. F., Partridge, J. G., Schulze, M., Scott, S. & Brown, S. A. Measurement of the Conductivity Exponent in Random Percolating Networks of Nanoscale Bismuth Clusters. in *Proceedings of the IEEE* 0–5 (2003).

[32] Kirkpatrick S (1973). Percolation and Conduction. Rev. Mod. Phys..

[33] Jensen P (1999). Growth of nanostructures by cluster deposition: Experiments and simple models. Rev. Mod. Phys..

[34] Gefen Y, Shih W-H (1986). Nonlinear Behavior near the Percolation Metal-Insulator Transition. Phys. Rev. Lett..

[35] Beloborodov IS, Lopatin AV, Vinokur VM (2007). Granular electronic systems. Rev. Mod. Phys..

[36] Sondheimer EH (1952). The mean free path of electrons in metals. Adv. Phys..

[37] Arnason SB, Herschfield SP, Hebard AF (1998). Bad Metals Made with Good-Metal Components..

[38] Voss RF, Laibowitz RB, Allessandrini EI (1982). Fractal (Scaling) Clusters in Thin Gold Films near the Percolation Threshold. Phys. Rev. Lett..

[39] Wegner K, Piseri P, Tafreshi HV, Milani P (2006). Cluster beam deposition: a tool for nanoscale science and technology. J. Phys. D. Appl. Phys..

[40] Lassesson A, Brown SA, Lith J, Van & Schulze M (2008). Electrical characterization of gold island films: A route to control of nanoparticle deposition. Appl. Phys. Lett..

[41] Creyssels M (2007). Some aspects of electrical conduction in granular systems of various dimensions..

[42] Duxbury PM, Beale PD, Leath PL (1986). Size Effects of Electrical Breakdown in Quenched random Media. Phys. Rev. Lett..

[43] Olsen M, Hummelgård M, Olin H (2012). Surface Modifications by Field Induced Diffusion. PLoS One.

[44] Elteto K, Antonyan EG, Nguyen TT, Jaeger HM (2005). Model for the onset of transport in systems with distributed thresholds for conduction. Phys. Rev. B - Condens. Matter Mater. Phys..

[45] Middleton AA, Wingreen NS (1993). Collective transport in arrays of small metallic dots. Phys. Rev. Lett..

[46] Parthasarathy R, Lin X-M, Jaeger HM (2001). Electronic Transport in Metal Nanocrystal Arrays: The Effect of Structural Disorder on Scaling Behavior. Phys. Rev. Lett..

[47] Sawa A (2008). Resistive switching in transition metal oxides. Nanotechnology.

[48] Kulkarni, A. D. Artificial Neural Networks for Image Understanding. (John Wiley & Sons, 1997).

[49] Gokmen T (2016). Acceleration of Deep Neural Network Training with Resistive Cross-Point Devices: Design Considerations. Front. Neurosci..

