# Supplementary material for: Facile fabrication of complex networks of memristive devices
Source: Sci Rep. 2017 Aug 11;7:7955. doi: 10.1038/s41598-017-08244-y (PMC5554187; doi:10.1038/s41598-017-08244-y)
Supplement: Supplementary file 1 — Supplementary Information [file 41598_2017_8244_MOESM1_ESM.doc]

**Supplementary information**

**Facile fabrication of complex networks of memristive devices**

Chloé Minnai^1^, Andrea Bellacicca^1^, Simon. A. Brown^2, *^ and Paolo Milani^1, #^

*^1^ CIMAINA and Dipartimento di Fisica, Università degli Studi di Milano, via Celoria 16, 20133 Milano, Italy.*

*^2^ The MacDiarmid Institute for Advanced Materials and Nanotechnology, Department of Physics and Astronomy, University of Canterbury, Private Bag 4800, Christchurch 8140, New Zealand.*

**Figure S1**. Typical variation of device resistance in response to applied voltages for devices with initial resistances ~50 kΩ (top panel) and ~10 kΩ (bottom panel) i.e. initial resistances higher and lower than that of the device shown in Fig. 1.


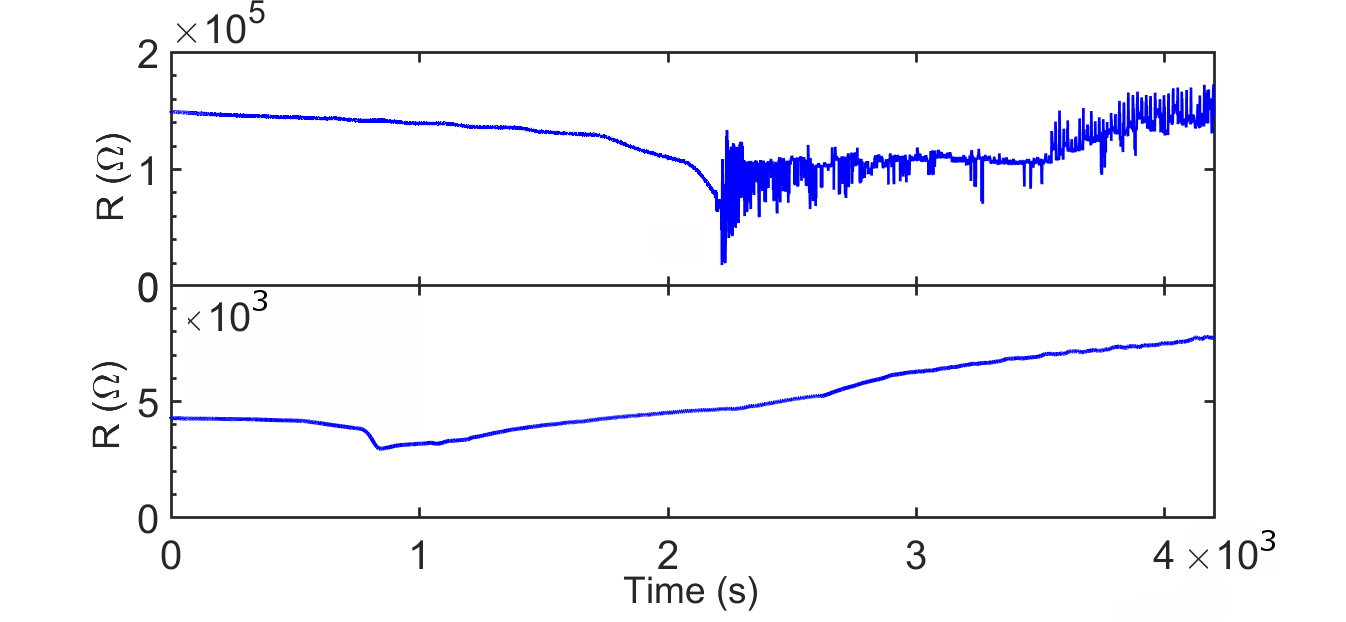


We fabricated devices with an initial resistance in the range 1 kΩ - 1 MΩ. Each of the samples was measured with the procedure described in the “Methods” section of the main text.

Different switching behaviour was found according to the initial resistance of the sample. Obviously different resistances correspond to different surface coverages of the deposited particles (see for example Refs 24 and 28).

Samples with a resistance below ~10 kΩ exhibit no switching events even under a bias of 60 V. Only a resistance evolution in response to the voltage applied is recorded as shown in the bottom panel of Fig. S1. We believe the resistance evolution is due to melting of critical connections in the films which causes either coalescence (early stages) or breaking of connections (later), and that the absence of discrete switching events is due to the higher coverage of particles and consequent increase in the number of parallel connections.

Samples with an initial resistance higher than ~ 50 kΩ exhibit a rich pattern of switching events, as shown in the top panel of Fig. S1. The activation process, in this case, occurs at high voltages (90-100 V). For this reason, in the main text, we focused on devices with an initial resistance in the range 10-50 kΩ, as they show complex pattern of switching events at a lower voltage.
